# Public health round-up

**DOI:** 10.2471/BLT.15.010715

**Published:** 2015-07-01

**Tribute to health workers in the Ebola epidemic**

Nursing students wait to take their final exams at the Connaught Hospital in Freetown, Sierra Leone. The hundreds of health workers who lost their lives on the front-line of the Ebola epidemic in West Africa were honoured with a moment of silence at this year’s World Health Assembly in May. This image and the cover photograph were part of an exhibition called Health Workers Count, organized by the Global Health Workforce Alliance and WHO Health Workforce Department.

http://www.who.int/workforcealliance/media/news/2015/photoexhib_hw_count

**Figure:**
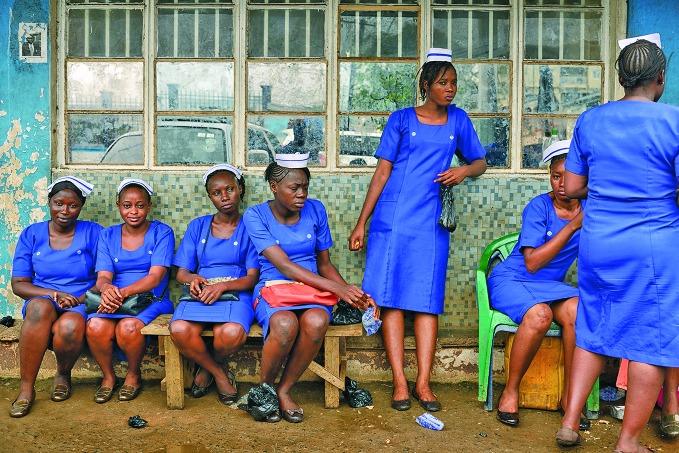
WHO/Samuel Aranda

## Nepal monsoon season

The World Health Organization (WHO) is deploying 50 medical camp kits, in collaboration with Nepal's health ministry and the World Food Programme, in the 14 worst earthquake-hit districts to ensure basic health services are restored before the onset of the monsoons. The kits comprise tents and other equipment to construct temporary health centres. 

As a result of two major earthquakes on 25 April and 12 May, 484 public health facilities were completely damaged and 474 partially damaged.

These medical camps are solar-powered and built to withstand conditions during the monsoon season.

“The medical camp kits will ensure continuity-of-care during the rainy season as there is no time to build permanent structures,” said Dr Frank Paulin, WHO's acting representative in Nepal.

Based on medical camp kits used in previous relief operations, the 50 kits have been developed in consultation with district health offices in Nepal and are tailored to the needs of the affected areas and populations.

The camps can be set up quickly to provide outpatient and inpatient facilities. The challenge is to keep them well stocked with medical supplies.

“These camps have been designed with the rainy season in mind, and they will be in use until at least the end of the monsoon.” said Michel Tomaszek, WHO’s chief in-country logistician.

WHO coordinated the foreign medical teams’ support to Nepal, to provide foreign health workers to work alongside Nepalese counterparts and attend to the health needs of the population in the worst affected areas.

Paulin said that their role in the aftermath of the disaster had helped to fill gaps in services, but that now the task was to restore and rebuild the health-care system.

http://www.who.int/emergencies/nepal/en/

## Sanitation targets lagging

The dire lack of sanitation particularly in Asia and sub-Saharan Africa should be an important part of the new Sustainable Development Goals (SDGs) when they are decided in September at the United Nations General Assembly, according to a report released last month.

The final report of the WHO/UNICEF Joint Monitoring Programme for Water Supply and Sanitation (JMP) summed up progress in water and sanitation throughout the whole period covered by the Millennium Development Goals (MDGs), from 1990 to 2015. 

It noted that the MDG target for safe drinking-water had been met in 2010 and that an estimated 91% of the world’s population uses an improved source of drinking water. 

But the MDG sanitation target is lagging badly behind and an estimated 2.4 billion people still have no access to improved sanitation facilities.

“We are particularly concerned by the fact that open defecation is still practised by an estimated 946 million people, including 610 million in South Asia alone and numbers still rising in sub-Saharan Africa,” said Dr Richard Johnston, technical officer for water, sanitation and hygiene at WHO. 

“At current rates of progress, open defecation will not be eliminated by 2030,” Johnston said, referring to the end of the next development goal phase. “Another major problem is inequality in access. While 82% of the urban population globally now uses improved sanitation facilities, only 51% of the rural population does.”

An estimated 842 000 deaths per year could be prevented by improving water, sanitation and hygiene in low- and middle-income countries. 

Post-2015 monitoring of the SDGs must go beyond the household and prioritize schools and health facilities, said the report. 

Monitoring should also address drinking-water quality, faecal waste management, and hand washing, which were not tracked in the MDGs, the report said. 

“The SDGs could be even more ambitious than the MDGs, given that universal coverage targets will require a much stronger focus on the people who have been left behind,” Johnston said. 

“At the same time, a greater emphasis on the safe management of faecal waste – which is included in the draft SDGs – will pose new challenges even for countries with high sanitation coverage.” 

“Countries will have to step-up the current rate of progress to reach the new SDG targets by 2030,” he said, referring to draft proposals for the SDGs that are due to be considered at the UN General Assembly in September. 

http://www.who.int/water_sanitation_health/en/

## MERS outbreak in Asia

Ten people died and 126 further cases were confirmed in an outbreak of Middle East respiratory syndrome coronavirus (MERS-CoV) in the Republic of Korea, as of 12 June.

It is the largest outbreak of MERS-CoV outside Saudi Arabia, since the disease was first identified in 2012.

WHO and the country’s health ministry launched a joint mission on 10 June in the capital, Seoul, comprising a team of experts in epidemiology, virology, clinical management, infection prevention and control, and public health officers.

The mission led by Dr Keiji Fukuda, WHO Assistant Director-General for Health Security, and Dr Jong-Koo Lee, Director of the Center for Global Medicine at Seoul National University, made several recommendations based on its initial findings.

These recommendations included strengthening of prevention and control measures in health facilities across the country and close investigation of patients who come to health facilities with fever or respiratory symptoms. 

It also recommended that close contacts of MERS-CoV cases should not travel while being monitored for the development of symptoms and that “strong consideration” should be given to re-opening schools, as they had not been linked to transmission of the virus.

“The joint mission is trying to gain a better understanding of transmission patterns and risk factors in this outbreak. It is also reviewing the public health measures implemented to date,” Fukuda said.

The country’s index (first) case was confirmed on 20 May. The person concerned had recently travelled to Bahrain, Qatar, Saudi Arabia and the United Arab Emirates.

Although he was not ill during his travels, he became symptomatic on return to the Republic of Korea and visited two outpatient health clinics and two hospitals seeking treatment.

Since the identification of the first laboratory confirmed case, the Korean authorities have been working hard to trace all possible contacts including health-care workers caring for the patient, other patients at the same health-care facilities and family members.

On 26 May, one person with a confirmed case of MERS-CoV travelled from the Republic of Korea to China. As of 3 June, 1369 contacts were being followed in quarantine or isolation at home or in state-run facilities.

http://www.wpro.who.int/mediacentre/releases/2015/201506010/en/

**Cover photo**

A health worker in full personal protective clothing waits in an ambulance in Sierra Leone.

**Figure:**
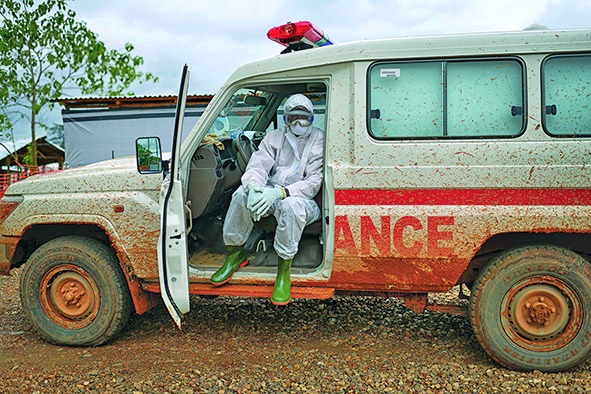
WHO/Samuel Aranda

## Heat waves and health

Countries need to prepare for more intense and frequent heat waves as a result of climate change as these can pose serious health risks and have other consequences, according to a joint publication released last month by WHO and the World Meteorological Organization (WMO).

The publication entitled *Heat waves and health: guidance on warning-system development* contains detailed guidance on how countries can establish heat–health warning systems. 

These systems provide meteorological and climate prediction-based information on the likelihood of the occurrence of hot weather, which can trigger heat rash, heat cramps, heat exhaustion, heatstroke and death.

The information generated by these early warning systems can be used by decision-makers to take measures to reduce the possible negative effects on people’s health and to alert the public to impending dangerous hot weather.

Hundreds of deaths in addition to those that would normally be expected were recorded during heat waves last month in India as well as in the Russian Federation in 2010 and France in 2003. 

Heat waves can place additional burdens on health and emergency services, strain energy and water supplies and affect transport systems.

“It is hoped that the guidance will act as a catalyst for bringing together key players from climate, health, emergency-response agencies and decision-makers, as well as the general public, for initiating action concerning the overall management of heat as a hazard,” said Maria Neira, director of the Department of Public Health, Social and Environmental Determinants of Health at WHO, and Maxx Dilley, director of the Climate Prediction and Adaptation Branch at WMO, in the publication’s foreword.

Heat waves are periods of unusually hot and dry, or hot and humid, weather that have a subtle onset and cessation. Heat waves last at least two to three days and usually have a discernible impact on human and natural systems, according to the guidance.

Heat waves are not defined by a specific temperature range and thus the same meteorological conditions can constitute a heat wave in one place but not in another.

http://www.who.int/globalchange/en/

**Looking ahead**

13 – 16 July – 3rd International Conference on Financing for Development. Addis Ababa, Ethiopia. http://www.un.org/esa/ffd/overview/third-conference-ffd.html

28 July – World Hepatitis Day.

25 – 27 September – United Nations Summit to adopt the post-2015 development agenda. New York, USA.

